# Willingness to be vaccinated against COVID-19 and associated factors in Migori County, Kenya: Analysis of cross-sectional observational survey data

**DOI:** 10.1371/journal.pgph.0003699

**Published:** 2025-03-13

**Authors:** Gianna Ferrara, Sandra Mudhune, Ash Rogers, Julius Mbeya, Alyn Achieng, Vincent Were, Constance Shumba, Alice Muga, Joseph Starnes

**Affiliations:** 1 Vanderbilt University School of Medicine, Master of Public Health Program, Nashville, Tennessee, United States of America; 2 Lwala Community Alliance, Rongo, Kenya; 3 Adaptive Model for Research and Empowerment of Communities in Africa, Kisumu, Kenya; 4 Research Division, Data Synergy and Evaluation Unit, African Population and Health Research Center (APHRC), Nairobi, Kenya; 5 Division of Epidemiology and Social Sciences, Institute for Health and Equity, Medical College of Wisconsin, Milwaukee, Wisconsin, United States of America; 6 Department of Health, Migori County, Kenya; 7 Department of Pediatrics, Division of Pediatric Cardiology, Vanderbilt University Medical Center, Nashville, Tennessee, United States of America; University of Embu, KENYA

## Abstract

**Introduction:**

The COVID-19 pandemic revealed the daunting challenge of vaccine hesitancy. We aimed to describe attitudes towards virus containment and vaccination in rural Kenya. Identifying factors associated with willingness to be vaccinated and attitudes towards information sources and health worker outreach, will allow for targeted programming and prevention methods.

**Methods:**

This was a cross-sectional observational survey. This study was conducted in Migori County, Kenya. 7,196 heads of households were surveyed between May 3, 2021 and June 25, 2021. The primary outcome was willingness to receive the COVID-19 vaccine.

**Results:**

5,386 of the 7,196 total heads of households (74.8%) were willing to get the COVD-19 vaccination. Those willing to get tested if experiencing COVID-19 symptoms (AOR=7.51, 95% CI=3.04-18.55, P-value<0.001) and those who believe everyone should be vaccinated according to the national vaccine schedule (AOR=18.91, 95% CI=6.76-52.88, P-value<0.001) were more likely to be willing to receive a COVID-19 vaccine. The recommendation of the Ministry of Health was the highest factor in willingness to be vaccinated, with 27% (1942) reporting this recommendation extremely influenced their decision. Nearly half of respondents (3047, 42.3%) believed there is a possibility that COVID-19 is a global conspiracy. None of the demographic factors analyzed were associated with willingness to get the vaccination.

**Discussion:**

We describe factors that contribute to willingness to get a new vaccination in a rural Kenyan community. Measuring vaccine willingness against covariables selected based on previous literature and programmatic experience provides hyper-local information to improve regional programming and future pandemic preparedness for organizations working in similar environments.

## Introduction

In order to combat a new virus in humans, epidemiologists work with officials to identify the source of the outbreak, monitor, and track the disease, study the disease, and develop guidance to slow the spread and lessen the impact [1]. In December 2019, severe acute respiratory syndrome coronavirus 2 (SARS-CoV-2) emerged, resulting in a respiratory illness called coronavirus disease 2019 (COVID-19). Symptoms, in those who exhibit them, appear between 2 and 14 days following exposure [2]. Those infected are contagious for up to two days before experiencing symptoms and remain contagious for 10 to 20 days after, thus allowing the virus to spread rapidly [2].

The COVID-19 pandemic has negatively impacted national economies, food systems, and the mental and physical well-being of people around the world. Countries, such as Kenya, that deal with socioeconomic struggles including food insecurity and ongoing public health issues, as well as a workforce mostly comprised of informal economy workers, were particularly exposed to the effects of the pandemic [2]. As of 2023, there were over 9 million cases of COVID-19 reported within the African continent. Over 175,000 deaths were reported; at least 5,600 of those were in Kenya [3]. Kenya has a population of about 50 million people, with about 75% living in rural areas [4]. Self-reported exposure to disinformation is apparent, as 2 in 3 Kenyans reported seeing stories they believed were untrue regarding COVID-19 [5]. Specifically, there was a high response of Kenyans reporting to have seen articles claiming the coronavirus was created and spread by China [5].

The development of a successful vaccine and timely vaccination rollout was essential in order to slow the ongoing global burden of the pandemic. Understanding and evaluating the willingness, or intent, to get vaccinated is important in improving uptake. Improved communication measures, among other strategies, play a significant role in combating a pandemic [6].

As of April 2021 when this study was conducted, 82% of COVID-19 vaccinations had been delivered to high- and upper-middle-income countries, while just 0.2% went to low- and middle-income countries (LMICs) [4,7]. The disparities between access to resources added to mistrust in healthcare systems and increased vaccine hesitancy among communities in LMICs, further exacerbating vaccine access inequity [8]. At the time the survey was administered, a COVID-19 vaccination was not readily available to the population; thus our study data pertains to attitudes towards a hypothetical vaccine.

Low COVID-19 vaccine uptake among health care providers, across 5 countries in Sub-Saharan Africa, was associated with concerns about the effectiveness of the vaccine, side-effects, and fear of receiving an experimental treatment [9]. The association between pre-existing conditions, participant trust, perceived personal risk, and perceived higher burden of COVID-19 and the adoption of precautionary measures, shows higher perceptions of personal risk are impacted by pre-existing health conditions and negative experiences with the virus in participants’ social networks [10]. Being male, having a higher level of education, and fear of contracting the virus are factors associated with positive attitudes towards the vaccine [11]. In LMICs specifically, vaccine hesitancy may be due to a lack of knowledge about vaccine effectiveness in reducing the burden, morbidity, and mortality of communicable disease [12]. The roll-out of Ebola vaccines in west Africa showed that the physical presence of the vaccine drives the involvement of national leadership, spreading to local communities [12]. The low vaccine acceptance rates (VARs) in Africa have immense global health implications. These low rates may facilitate the emergence of immune invading SARS-CoV-2 variants of concern (VOCs) that can spread across countries [13].

In 2007, the Lwala Community Alliance (Lwala) was established to promote the health and well-being of rural communities in Kenya. The organization operates a hospital with inpatient, outpatient, maternal, child, reproductive, and HIV clinics in North Kamagambo in northeast Migori County. In addition, Lwala partners with the Kenya Ministry of Health to strengthen community governance in healthcare, support cadres of paid, trained, supervised, and supplied community health workers, and improve the quality of services at health facilities. [14].

This study will provide insight on the willingness to be vaccinated against COVID-19 and associated factors among the general population in a Kenyan community, focusing on the adherence to and the adoption of containment measures. The variety of data included in this study, such as measures taken, knowledge of someone dying as a result of COVID-19, and sources of information used for pandemic related news were examined in association with one’s willingness to receive a not yet readily available vaccination. Most published literature studies healthcare providers or populations recruited at healthcare facilities, where bias may be present. Our study does not focus on a specific occupational population.

## Materials and methods

### Study setting

**Fig 1:**
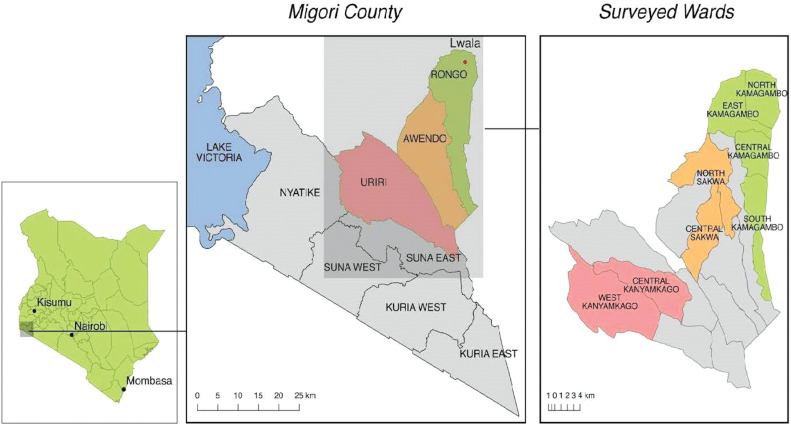
Migori County, Kenya. Lwala programming began in North Kamagambo in Rongo sub-county (green). All of Rongo, with the exception of Central Kamagambo, received Lwala COVID-19 services at the time the survey was conducted. All Community Health Workers (CHWs) in Rongo received up-to-date training. Wards in Uriri (West Kanyamkago and Central Kanyanmkago) serve as comparison wards (red). Awendo was planned to receive programming next (orange). Central Kamagambo received programming later in 2021.

Migori County is one of 47 counties in Kenya, located in the former Nyanza province, with a population of around 1.1 million [15]. The county’s economy primarily depends on subsistence farming as well as fishing in areas bordering Lake Victoria. This region has historically underperformed on health metrics and has some of the poorest health outcomes in Kenya [14,16]. The 2021 Lwala Household Survey (May 3, 2021 - June 25, 2021) was used to obtain data on residents of Rongo, Awendo, and Uriri sub-counties in Migori County, Kenya as a part of an ongoing repeated cross-sectional study [Fig 1].

### COVID-19 in migori county

During COVID-19, Lwala partnered with Migori County to implement strategies and protocols pertaining to screening, infection control, treatment, sanitation, nutrition, education, and social support. In the midst of the pandemic, Migori County faced shortages of COVID-19 PCR test kits and long delays in receiving PCR results. Community Health Workers (CHWs) played a critical role, following a protocol for COVID-19 and remaining involved in the decision-making process as the pandemic continued. Lwala created and repeatedly updated their COVID-19 response plan in response to the Kenya Ministry of Health, World Health Organization, and Africa CDC guidance, including the suspension of in-person gatherings, encouragement of hand washing, personal protective equipment (PPE) use, and early ordering of extra stocks of drugs and other commodities. As CHWs are an important asset in Lwala, they were trained in small groups to provide education, sensitization, and surveillance and to continue the provision of primary health services. A protocol specific to CHWs was developed to ensure community members continued to receive community-based preventative and curative care with a focus on maternal and child health services. By June 2021, Migori county had provided over 13,200 frontline workers and eligible people with one vaccine dose. Of this population, over 80% of CHWs had received a vaccination [17]. However, as of June 2021, only about 2% of Migori County’s population was vaccinated against COVID-19 [17].

To monitor impact and allow for improvement, a repeated cross-sectional household survey was designed to assess a wide variety of health metrics in both Lwala programming and control areas [14,18–21]. This study utilizes the 2021 survey, with data from May 3 through June 25, to assess COVID-19 vaccine hesitancy and pandemic experience in Migori County. Through understanding the pandemic experience of Migori County residents and their hesitancy towards receiving a COVID-19 vaccination, our study will provide data for programming and may also be helpful for those working in similar settings.

### Sampling and survey

This study is part of a repeated cross-sectional survey that will be used to evaluate key health metrics in Lwala Community Alliance supported areas in comparison to control areas in Migori County. The main objectives of the survey include assessing the health, socioeconomic, and education status of current and future communities receiving Lwala programming [18].

The survey methods have been described in detail elsewhere [18]. Briefly, data collection began in 2021 and will occur triennially until 2027. The sample size was calculated to detect a 10% change in health metrics over time. This yielded a sample size of at least 621 households per area, which was inflated by 30% to give a total target sample of 887 per area (7,096 total households) [18]. Households were selected using a procedure based on the World Health Organization Expanded Programme of Immunization (EPI) method [22]. Geographic Information System (GIS) technology was used to split areas into 127 grid squares and calculate the center points of each square, which served as the starting point for surveying. The spin-the-bottle method was then used to select households. Trained enumerators hired from the community administered the surveys in person, under the guidance of an overall survey supervisor [18]. This study utilizes data from the 2021 survey, which included 7,196 households. Prior to data collection, enumerators obtained informed consent from participants through signatures or thumbprints. Data were accessed between July 11, 2023 and February 15, 2024 for analysis.

Vaccine willingness was defined as responding “yes” to willingness to receive a COVID-19 vaccination. Analyzed covariates were selected from the 2021 Household Survey responses based on previous literature and programmatic experience. Demographic variables pertaining to COVID-19 views and practices as well as sources used to find information on COVID-19 were extracted.

### Statistical methods

Data management and analysis was conducted using STATA version 15 (StataCorp LP, College Station, TX). The outcome variable was “when the vaccine is available to you, will you get it?” The variable was coded as “yes” or “no”. The independent variables included age (18-30, 31-40, 41-50, above 50), sex (male, female), marital status (married/cohabiting, unmarried), education level (primary or less, secondary or more), household size (<=4, 5-9,>=10), main source of income (employment, casual labour, agriculture, self-employed), household region (North Kamagambo, East Kamagambo, Central Kamagambo, South Kamagambo, Central Kanyamkago, West Kanyamkago, North Sakwa and Central Sakwa), fertility preference (wanted another child, no more, cannot get pregnant, undecided), self-report of previous COVID infection (yes, no), know someone dead from COVID (yes, no), consider self at risk of COVID (yes, no), own face mask or covering (yes, no), believe COVID is global conspiracy (yes, no), testing if you have COVID symptoms (yes, no), everyone should be vaccinated according to national vaccine schedule (yes, no), believe vaccine can help control spread (yes, no, not sure), if had COVID-19 prior would not get vaccine (yes, no, not sure), if everyone else is vaccinated I do not need (yes, no, not sure), and visited by a CHW in the past 3 months (yes, no).

Descriptive analyses were conducted, and frequencies and percentages were reported. Multivariable logistic regression was conducted to determine factors that influence whether a person would get the COVID-19 vaccine if available.

### Ethical approval

The study was approved by the Ethics and Scientific Review Committee at AMREF Health Africa (AMREF-ESRC P452/2018) and the Institutional Review Board at Northeastern University (IRB #: 20-09-18). A research permit was obtained through the National Commission for Science, Technology, and Innovation in Kenya (NACOSTI/P/21/8776). Informed written consent was obtained from all participants prior to survey administration. This study used deidentified, retrospective data from a previously administered household survey.

## Results

**As shown in** Table 1, 5,386 of the 7,196 total heads of household (74.8%) were willing to get the COVID-19 vaccination. The majority of respondents were aged 18-30 years (59.9%), were female (87.4%), were married/cohabiting (84.9%), had primary education or less (53.1%), had a household size of <=4 (63.9%), and were casual labourers (31.5%). In terms of fertility preference, most respondents wanted another child (51.8%). Respondents from North Kamagambo had the highest willingness to receive the vaccine at 78.0%.

**Table 1:** Demographics by willingness to get COVID Vaccine.

Willing to receive vaccine	No (n=1810)	Yes (n=5386)	Total (n=7196)
	1810(25.2)	5386(74.8)	7196(100)
**Characteristics**	**n(%)**	**n(%)**	**n(%)**
**Age**			
18-30	1099(25.5)	3213(74.5)	4312(59.9)
31-40	510(25.3)	1505(74.7)	2015(28.0)
41-50	108(21.5)	395(78.5)	503(7.0)
Above 50	93(25.4)	273(74.6)	366(5.1)
**Sex**			
Female	1600(25.4)	4690(74.6)	6290(87.4)
Male	210(23.2)	696(76.8)	906(12.6)
**Marital Status**			
Married/cohabiting	1506(24.7)	4599(75.3)	6105(84.9)
Unmarried	303(27.8)	787(72.2)	1090(15.2)
**Education Level**			
Primary or less	986(25.8)	2837(74.2)	3823(53.1)
Secondary or more	823(24.4)	2548(75.6)	3371(46.9)
**Household size**			
<=4	1212(26.4)	3384(73.6)	4596(63.9)
5-9	586(23.0)	1964(77.0)	2550(35.4)
>=10	12(24.0)	38(76.0)	50(0.7)
**Main source of income**			
Employment	316(20.5)	1227(79.5)	1543(21.6)
Casual labour	592(26.3)	1656(73.7)	2248(31.5)
Agriculture	462(29.1)	1128(70.9)	1590(22.3)
Self-employed	383(23.7)	1230(76.3)	1613(22.6)
Other	36(26.5)	100(73.5)	136(1.9)
**Household Region**			
North Kamagambo	197(22.0)	697(78.0)	894(12.4)
East Kamagambo	237(26.2)	669(73.8)	906(12.6)
South Kamagambo	278(31.3)	609(68.7)	887(12.3)
Central Kamagambo	217(22.9)	732(77.1)	949(13.2)
Central Kanyamkago	207(23.6)	671(76.4)	878(12.2)
West Kanyammkago	209(23.1)	697(76.9)	906(12.6)
North Sakwa	248(26.7)	681(73.3)	929(12.9)
Central Sakwa	217(25.6)	630(74.4)	847(11.8)
**Fertility preference**			
Want another child	830(22.3)	2889(77.7)	3719(51.8)
No more	458(23.9)	1456(76.1)	1914(26.7)
Cannot get pregnant	97(32.2)	204(67.8)	301(4.2)
Undecided	421(33.8)	826(66.2)	1247(17.4)

Table 2 **shows** the vast majority of participants had not been infected with COVID-19 as of June 25, 2021 (99.2%), but most considered themselves at risk of COVID-19 infection (81.5%). The majority of respondents owned face masks or coverings (94.9%) and would be tested if experiencing COVID-19 symptoms (77.4%). Most participants who had a prior COVID-19 diagnosis at the time of the survey would be willing to be vaccinated (77.3%). Nearly half of respondents believe there is a possibility COVID-19 is a global conspiracy (42.3%). “Everyone should be vaccinated according to the national vaccine schedule” was a common belief for those who were willing to be vaccinated for COVID-19 (89.9%). Most respondents disagreed with the statement that “if everyone else is vaccinated, they do not need to be” (88.2%).

**Table 2:** Attitude/perception towards COVID.

Willing to receive vaccine	No (n=1810)	Yes (n=5386)	Total (n=7196)
**Characteristics**	**n(%)**	**n(%)**	**n(%)**
**Previously infected with COVID**			
Yes	13(22.8)	44(77.2)	57(0.8)
No	1794(25.2)	5322(74.8)	7116(99.2)
**Know someone dead from COVID**			
Yes	24(19.8)	97(80.2)	121(30.5)
No	69(25.0)	207(75.0)	276(69.5)
**Consider self-risk of COVID**			
Yes	1594(27.2)	4266(72.8)	5860(81.5)
No	216(16.2)	1119(83.8)	1335(18.5)
**Own face mask or covering**			
Yes	1645(24.2)	5141(75.8)	6786(94.9)
No	156(42.6)	210(57.4)	366(5.1)
**Believe COVID is a global conspiracy**			
Yes	641(21.0)	2406(79.0)	3047(42.3)
No	612(21.9)	2178(78.1)	2790(38.8)
Maybe	557(41.1)	799(58.9)	1356(18.9)
**Testing if you have COVID symptoms**			
Yes	845(15.2)	4708(84.8)	5553(77.4)
No	960(59.1)	664(40.9)	1624(22.6)
**Everyone should be vaccinated according to national vaccine schedule**			
Yes	583(10.1)	5185(89.9)	5768(80.2)
No	1226(86.0)	199(14.0)	1425(19.8)
**Believe vaccine can help control spread**			
Yes	511(10.1)	4540(89.9)	5051(70.2)
No	1298(60.5)	846(39.5)	2144(29.8)
**If had prior COVID-19 infection**			
Would not get vaccine	423(39.1)	660(60.9)	1083(15.1)
Would get vaccine	1386(22.7)	4724(77.3)	6110(84.9)
**If everyone else is vaccinated, I do not need**			
Yes	388(45.5)	464(54.5)	852(11.8)
No	1422(22.4)	4921(77.6)	6343(88.2)

**Fig 2:**
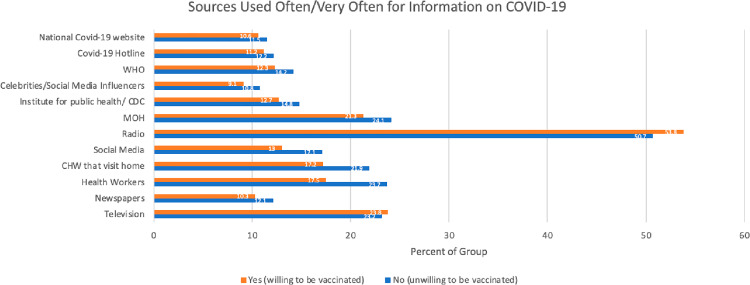
Highlights sources of information used for information on COVID-19. Radio was the most used source of information regarding COVID-19, with 53% saying they used it often or very often. The majority of respondents fell in the sometimes category for all sources, aside from the majority responding often or very often to the use of radio. Newspapers were the least used information source, with 10.7% reporting they used it often or very often, followed by celebrities/social media, with 14.1% saying they used it often or very often. The Ministry of Health (21.3%), health workers (17.5%), and the CHW that visits home (17.2%) were used very often by participants, notably more often than both social media and newspapers (Fig 2).

The recommendation of the Ministry of Health was the largest factor in vaccine decision making, on both ends of willingness, with 21.3% who were unwilling to receive a vaccine and 28.9% who were willing to saying this recommendation extremely influenced their decision, **as seen in** Table 3. Whether the vaccine has been in use for a long time with no side effects (26.2% responding extremely influential) and whether the vaccine is free (25.3% reporting extremely influential) both played large roles in respondents’ decision making. The country in which the vaccine is produced was the least cited factor in COVID-19 decision making, with 13.2% saying this extremely influenced their decision. The majority of respondents fell in the slightly/somewhat/moderately category for all statements. Recommendation from the family doctor/healthcare provider (62.5%) and recommendation from a CHW visiting the home (64.4%) were moderately considered as a factor in willingness to get vaccinated. The impact vaccination uptake would have on the lifting of restrictions on movement and group gatherings was an influential factor for the majority of all participants, whether they were willing or unwilling to receive the vaccine (18.5% reporting extremely, 65.2% reporting moderately).

**Table 3:** COVID-19 vaccine decision factors.

Willing to receive vaccine	No (n=1810)			Yes (n=5386)			Total (n=7196)		
	Not at all	Slightly/ Somewhat/ Moderately	Extremely	Not at all	Slightly/ Somewhat/ Moderately	Extremely	Not at all	Slightly/ Somewhat/ Moderately	Extremely
Country in which the vaccine is produced	377(20.8)	1147(63.4)	285(15.8)	1366(25.4)	3354(62.4)	659(12.3)	1744(24.3)	4503(62.6)	947(13.2)
Recommendation from my family doctor/healthcare provider	306(16.9)	1209(66.8)	294(16.3)	1014(18.9)	3285(61.1)	1077(20.0)	1321(18.4)	4496(62.5)	1374(19.1)
Recommendation from a Community Health Worker that visits my home	305(16.9)	1192(65.9)	311(17.2)	882(16.4)	3437(63.9)	1058(19.7)	1187(16.5)	4632(64.4)	1372(19.1)
Recommendation of the Ministry of Health (MOH)	299(16.5)	1125(62.2)	385(21.3)	737(13.9)	3093(57.5)	1553(28.9)	1036(14.4)	4220(58.6)	1942(27.0)
The track record of the vaccine’s use	279(15.4)	1153(63.7)	378(20.9)	782(14.5)	3097(57.5)	1504(27.9)	1062(14.8)	4253(59.1)	1884(26.2)
Whether the vaccine is used in other countries	294(16.3)	1220(67.5)	293(16.2)	913(17.0)	3357(62.4)	1108(20.6)	1208(16.8)	4580(63.7)	1403(19.5)
Risk of getting infected with COVID-19 at the time when the vaccine is available	279(15.5)	1191(66.0)	336(18.6)	786(14.6)	3266(60.8)	1322(24.6)	1066(14.8)	4459(62.1)	1661(23.1)
How easy it is to access	268(14.8)	1208(66.8)	332(18.4)	730(13.6)	3209(59.7)	1424(26.7)	999(13.9)	4419(61.5)	1769(24.6)
Whether vaccine is free	273(15.1)	1175(65.0)	360(19.9)	716(13.3)	3207(59.6)	1454(27.0)	990(13.8)	4384(61.0)	1817(25.3)
Whether a high vaccination uptake would lift restrictions on movement and gathering in groups	283(15.7)	1217(67.3)	308(17.0)	889(16.6)	3462(64.5)	1020(18.9)	1172(16.3)	4682(65.2)	1331(18.5)

**As shown in** Table 4, respondents who were willing to be tested if they had COVID symptoms (AOR=6.86, 95% CI=2.60-18.09, P-value<0.001) and those who thought that everyone should be vaccinated according to the vaccine schedule (AOR=18.25, 95% CI=5.82-57.22, P-value<0.001) were more likely to be willing to receive COVID-19 vaccination if available to them. Respondents who agreed that if they had COVID prior they would not get the vaccine (AOR=0.28, 95% CI=0.08-0.95, P-value=0.042) and those who were not sure (AOR=0.10, 95% CI=0.02-0.43, P-value=0.002) were less likely to be willing to receive COVID-19 vaccination if available to them compared to those who disagreed. Ever visited by a community health worker within the past 3 months, was also not included due to causing collinearity.

**Table 4:** Willingness to receive a COVD-19 vaccination - a regression analysis.

Characteristics	AOR(95% CI)	P-value
**Age**		
18-30	Ref	Ref
31-40	1.62(0.55-4.74)	0.379
41-50	1.08(0.17-6.66)	0.936
Above 50	3.03(0.22-42.66)	0.411
**Sex**		
Female	Ref	Ref
Male	0.76(0.23-2.56)	0.661
**Marital Status**		
Married/cohabiting	1.16(0.30-4.48)	0.826
Unmarried	Ref	Ref
**Education Level**		
Primary or less	Ref	Ref
Secondary or more	0.55(0.21-1.49)	0.242
**Household size**		
<=4	Ref	Ref
5-9	0.80(0.27-2.35)	0.683
>=10	1.89(0.01-604.96)	0.829
**Main source of income**		
Employment	Ref	Ref
Casual labour	0.60(0.16-2.19)	0.434
Agriculture	0.31(0.07-1.39)	0.126
Self-employed	0.71(0.21-2.44)	0.583
Other	0.20(0.01-4.15)	0.296
**Household Region**		
North Kamagambo	Ref	Ref
East Kamagambo	1.52(0.34-6.83)	0.585
Central Kamagambo	0.41(0.09-1.91)	0.258
South Kamagambo	0.41(0.09-1.91)	0.259
Central Kanyamkago	2.14(0.33-14.03)	0.424
West Kanyamkago	0.83(0.12-5.59)	0.85
North Sakwa	0.98(0.17-5.66)	0.978
Central Sakwa	3.63(0.61-21.38)	0.155
**Fertility preference**		
No more	Ref	Ref
Want another child	1.43(0.45-4.49)	0.545
Cannot get pregnant	2.49(0.01-496.36)	0.736
Undecided	1.32(0.26-6.64)	0.732
**Infected with COVID**		
Yes	1.73(0.28-10.59)	0.551
No	Ref	Ref
**Know someone dead from COVID**		
Yes	1.41(0.52-3.88)	0.5
No	Ref	Ref
**Consider self-risk of COVID**		
Yes	1.22(0.39-3.86)	0.736
No	Ref	Ref
**Believe COVID is global conspiracy**		
Yes	1.37(0.52-3.62)	0.529
No	Ref	Ref
Maybe	0.99(0.27-3.67)	0.993
**Own a face mask or covering**		
Yes	0.11(0.01-8.41)	0.319
No	Ref	Ref
**Testing if you have covid symptoms**		
Yes	6.86(2.60-18.09)	**<0.001**
No	Ref	Ref
**Everyone should be vaccinated according to national vaccine schedule**		
Yes	18.25(5.82-57.22)	**<0.001**
No	Ref	
**Believe vaccine can help control spread**		
Yes	3.43(0.90-13.13)	0.072
No	Ref	
Not sure	0.57(0.12-2.79)	0.486
**If had COVID-19 prior-would not get vaccine**		
Yes	0.28(0.08-0.95)	**0.042**
No	Ref	Ref
Not sure	0.10(0.02-0.43)	**0.002**
**If everyone else is vaccinated, I do not need**		
Yes	0.87(0.22-3.45)	0.840
No	Ref	Ref
Not sure	0.90(0.23-3.49)	0.875
**Source of information**		
**TV**		
Yes	0.90(0.30-2.68)	0.854
No	Ref	Ref
**Newspaper**		
Yes	1.11(0.23-5.34)	0.897
No	Ref	Ref
**Health Workers**		
Yes	0.62(0.19-2.08)	0.443
No	Ref	Ref
**CHW**		
Yes	0.39(0.11-1.41)	0.152
No	Ref	Ref
**Social Media**		
Yes	1.15(0.32-4.13)	0.835
No	Ref	Ref
**Radio**		
Yes	1.56(0.60-4.03)	0.357
No	Ref	Ref
**Ministry of Health**		
Yes	0.77(0.21-2.93)	0.706
No	Ref	Ref
**CDC**		
Yes	0.35(0.04-3.43)	0.370
No	Ref	Ref
**Celebrities**		
Yes	0.34(0.04-2.58)	0.295
No	Ref	Ref
**WHO**		
Yes	6.32(0.59-69.08)	0.131
No	Ref	Ref
**COVID Hotline**		
Yes	3.00(0.30-30.12)	0.351
No	Ref	Ref
**COVID Info Website**		
Yes	3.11(0.28-34.24)	0.354
No	Ref	Ref

*Values in bold are statistically significant.

*AOR-Adjusted Odds Ratio.

## Discussion

We find that the majority (74.8%) of residents in Migori County, a rural county in Kenya, would be willing to receive a COVID vaccine if available. This far exceeded the known availability of vaccines at the time of the survey. At this point, Kenya faced the global issue of inequality in access to vaccines. Lwala supported the Ministry of Health to vaccinate frontline health workers, with CHWs as a priority group. The majority of Lwala staff were vaccinated at a time when only 2% of the Kenyan population was vaccinated [17].

Willingness to be vaccinated was associated with specific aspects of the vaccine rollout. Participants reported whether the vaccine has been in use for an extended period of time with no side effects had the greatest impact on their decision to get vaccinated. This was consistent with findings among studies conducted in Sub-Saharan Africa showing the nature and degree of side effects was a significant factor influencing vaccine hesitancy [23].

Whether the vaccine is free and easy to access impacted respondents’ willingness to be vaccinated. This is consistent with findings across the African continent showing the significance of cost in the COVID-19 vaccination decision making process [24]. The decision factors, side effects, and cost emphasize the importance of both accurate information regarding vaccine side effects as well as governmental and non-governmental funding of vaccine efforts to improve uptake.

Radio was established as the most frequently used source of information (53%) regarding COVID-19 and may be the most effective way to spread accurate information about infectious diseases and vaccines in this setting. In 2021, the Media Council of Kenya reported that 74% of media consumption was through listening to the radio, followed by 58% watching television, and 25% reading newspapers within a majority rural population [25]. Radio was the most common source of information in our population, which is consistent among people living in the region both prior to the COVID-19 pandemic and during, while social media was the least common [26,27]. The Ministry of Health, health workers, and the CHW that visits home were used more often than both social media and newspapers. Those who used celebrities as a source of information on COVID-19 trended toward less likelihood of being vaccinated. These findings are helpful both for ongoing COVID-19 vaccination efforts and future epidemics.

A high percentage of respondents believed COVID-19 could be a global conspiracy. Skepticism about the vaccine in Kenya has been linked to disinformation and misinformation spread through communication methods, specifically social media [5]. Our population was limited to rural communities, where social media was the least reported form of communication used to access information on the pandemic and vaccine. This suggests misinformation may have been spread through local radio and newspapers, as these sources were reportedly accessed the most by participants. Distrust in the COVID-19 vaccine was stronger among those living in urban areas [5]. Given the rural nature of the households surveyed, it is consistent with published literature that willingness to be vaccinated may be higher in our population in comparison to more urban neighborhoods as willingness and skepticism go hand in hand.

The data shows the majority of respondents had not been infected with COVID-19 at the time of the survey. This may represent a truly low infection rate in a rural community. This could also be a result of lack of testing in the early days of the pandemic, due to asymptomatic cases not seeking testing, low inventory of tests available, or travel restrictions. Access to testing and health literacy are significant factors in regard to willingness to get tested when experiencing symptoms [29]. At the time of the survey, it was reported that Kenya tested an estimated 1 in every 10,000 inhabitants daily for active COVID-19 infections, which was 1/10 of the rate in the more developed countries of Spain and Canada [28]. Lwala programming data found increased positivity rates in April 2021 (Q2) not only due to increased testing but due to the increased severity and spread. Similarly, the majority of respondents did not know anyone who had died as a result of COVID-19. Importantly, the survey was conducted prior to the largest, most serious wave of COVID-infections in the community, with the prevalence of the Delta variant and more severe symptoms, which may have played a significant role in the low infection rate seen among the respondents.

Health behaviors of testing for the virus, wearing a mask, and willingness to be vaccinated ran together. Those willing to get tested if they experienced COVID-19 symptoms were more likely to be willing to get vaccinated. A trust in science and manufacturers behind COVID-19 tests, as well as in the health system and government guidance, may be a predictor for willingness to receive a vaccine. Recommendation from the family doctor/healthcare provider (62.5%) and recommendation from a CHW visiting the home (64.4%) were moderately considered as a factor in willingness to get vaccinated. The recommendation of the Ministry of Health was seemingly the largest factor in willingness to be vaccinated, with 27.0% saying this recommendation extremely influenced their decision. Similar results were found in a student population in Uganda [23].

Similarly, those who believed everyone should be vaccinated according to the national vaccine schedule had much higher odds of willingness to get the COVID-19 vaccine. This is not surprising as vaccine hesitancy was among the top 10 threats to global health per the World Health Organization in 2019, prior to the COVID-19 pandemic [30]. Those skeptical of vaccines that were developed slower and for familiar infectious diseases are more likely to be hesitant of COVID-19 vaccination. This behavior of overall vaccine hesitancy can be a predictor for one’s willingness to receive the COVID vaccine. This also emphasizes the need for long-term programming addressing underlying, more general vaccine hesitancy to allow for more rapid implementation of new vaccines during pandemic emergencies.

A significant minority in our population (1810, 25.5%) would be unwilling to get the vaccine, leaving substantial room for improvement in vaccine confidence. Previous studies have found that the lack of transparency in the use of COVID donations and funds negatively impacted the public’s view of its government in Kenya [31,32].

Despite a relatively high suspicion of COVID-19 being a global conspiracy, willingness to receive the vaccine among those who were skeptical remained high. Published literature shows that conspiracy theories are related to lower rates of vaccine acceptance [33,34,35,36]. Gaining public trust through health communications is key in improving vaccine uptake.

### Limitations

The survey is cross-sectional allowing for the study of association but does not allow for comment on causal relationships. Additionally, when the survey was administered a COVID-19 vaccination was not readily available to the population; thus our study data pertains to attitudes towards a hypothetical vaccine. This may explain the high rates of willingness to receive the vaccine, and we were not able to measure actual vaccination behavior. Furthermore, we evaluated the intent of respondents which may not align with behavior. Survey bias is possible as respondents must recall which information sources are most used to learn about COVID-19. However, this recall bias is unlikely to have a large effect on the results as they are current behaviors at the time of the survey. Lastly, the possible qualitative reasons to understand in-depth why 25.5% of our population would be unwilling to be vaccinated were not investigated.

## Conclusions

We describe factors that are associated with willingness to get an initial COVID-19 vaccination. Participants reported whether the vaccine has been in use for an extended period of time with no side effects and whether the vaccine is free as 2 main factors having the greatest impact on their decision to get vaccinated. Health behaviors of testing for the virus and willingness to be vaccinated ran together, as well as attitudes towards the national vaccine schedule and willingness to receive the COVID vaccine. Understanding the determinants of willingness to receive a new vaccination is key in the development of evidence-based messaging to encourage uptake. Further qualitative research on vaccine hesitancy among Kenyans, specifically in rural areas where barriers are greatest, is required to mitigate these challenges [4].
